# Neuropilin-1-mediated pruning of corticospinal tract fibers is required for motor recovery after spinal cord injury

**DOI:** 10.1038/s41419-019-1338-2

**Published:** 2019-01-25

**Authors:** Toru Nakanishi, Yuki Fujita, Toshihide Yamashita

**Affiliations:** 1 Department of Molecular Neuroscience, Graduate School of Medicine, Osaka University, Osaka, Japan; 2 WPI Immunology Frontier Research Center, Osaka University, Osaka, Japan; 3 Graduate School of Frontier Bioscience, Osaka University, Osaka, Japan; 4 Department of Neuro-Medical Science, Graduate School of Medicine, Osaka University, Osaka, Japan

## Abstract

Following incomplete spinal cord injury (SCI), reorganization of the corticospinal tract (CST) contributes to spontaneous motor recovery. Axotomized CST fibers form collaterals and make synapses with interneurons, followed by pruning of excess fibers. Although axonal pruning is involved in refinement of neural circuits, its molecular mechanisms and functional roles remain poorly understood. To address these questions, we performed dorsal hemisections of mouse thoracic spinal cord. We observed that *Neuropilin-1* (*Nrp1*) mRNA was upregulated in layer 5 pyramidal neurons in the motor cortex 14 days after SCI, when the pruning occurred. *Nrp1* knockdown using adeno-associated virus (AAV) vector encoding Nrp1 shRNA in the hindlimb motor area impaired the pruning of collaterals after SCI. Nrp1 knockout by injecting AAV vector encoding Cre recombinase into Nrp1 floxed mice also suppressed axonal pruning. Propriospinal neurons, interneurons that connect CST and motoneurons, expressed Semaphorin 3A (Sema3A), the ligand of Nrp1. Furthermore, the genetic deletion of Nrp1 specifically in the hindlimb motor area suppressed the recovery of skilled movement at 21 and 28 days after SCI. The present findings demonstrate that the pruning of collaterals mediated by Nrp1 is required for motor recovery after SCI, and suggest that refinement of the neuronal network facilitates motor recovery.

## Introduction

While complete spinal cord injury (SCI) often leads to permanent motor, sensory, and autonomic disorders, partial recovery of motor function is occasionally observed after incomplete SCI^1,2^. Recent studies suggest that motor recovery after SCI is attributable to the reorganization of damaged or undamaged descending pathways^3^, particularly the corticospinal tract (CST), which regulates voluntary movement^4^. In the course of CST reorganization, axotomized CST fibers form collaterals and make synapses with interneurons, such as propriospinal neurons above the injured site to construct compensatory neural pathways for bypassing the lesion^5–8^. Subsequently, excess collaterals are eliminated, a process called axonal pruning^6,8^. Although many studies have shown that axonal sprouting is beneficial for motor recovery^9^, the functional significance of pruning in motor recovery after SCI remains poorly understood. Pruning is widely observed in the developing brain, and is considered essential for activity-dependent elaboration of neural circuits^10,11^. Therefore, we investigated if axonal pruning was also necessary for motor recovery after SCI.

Here, we identified Neuropilin-1 (Nrp1) as a molecule responsible for axonal pruning. We further revealed that the pruning of collaterals was required for motor recovery after SCI. These findings may lead to the development of novel treatment for SCI. Specifically, the present study suggests that promoting the pruning of collaterals could facilitate motor recovery after SCI through the refinement of compensatory neural circuits.

## Results

### Pruning of collaterals occurs from 10 to 28 days after SCI

**Fig. 1:**
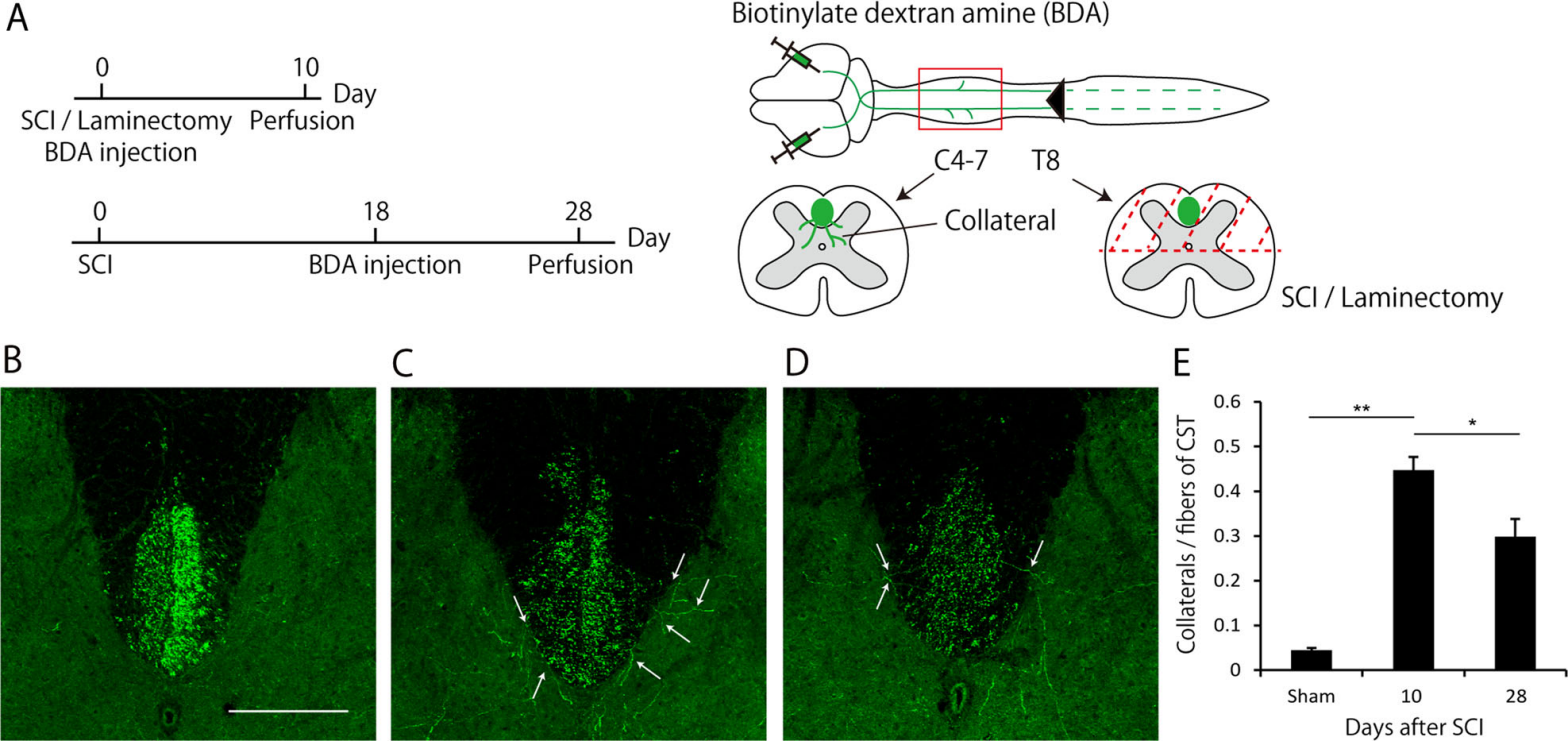
Pruning of collaterals occurs between 10 and 28 days after spinal cord injury (SCI). **a** Experimental scheme. Dorsal hemisection or laminectomy alone was conducted at T8 level in mice. An anterograde tracer, biotinylated dextran amine (BDA), was then injected into the hindlimb motor area to label the collaterals from corticospinal tract (CST). Mice were perfused at 10 or 28 days after SCI. The number of collaterals at the cervical cord (C4-7) was histologically assessed. **b-d** Transverse cervical sections showing BDA-labeled CST axons (green) in sham mice (**b**) and injured mice at 10 days (**c**) and 28 days (**d**) after SCI. White arrows indicate collaterals sprouted from CST. Scale bar: 200 μm. **e** Quantification of the number of collaterals. The total number of collaterals from C4 to C7 was divided by the number of main CST fibers running in the dorsal column. It was increased at 10 days after SCI and decreased at 28 days after SCI. Data are presented as mean ± SEM. *n* = 3–6, **p < 0.01,*p < 0.05, one way ANOVA followed by Tukey-Kramer test

We performed dorsal hemisections of the spinal cord of adult mice at thoracic level 8 (T8) (*n* = 12). As sham controls, we conducted only laminectomy at T8 of mice (*n* = 3; Fig. 1a). In this SCI model, main dorsal CST and minor dorsolateral CST were completely disrupted, whereas minor ventral CST remained intact^8,12^. As most CST fibers descend the dorsal funiculus^13^, hindlimb movement was impaired after dorsal hemisections.

The anterograde tracer BDA was injected into the hindlimb motor area bilaterally to visualize the collaterals from dorsal CST according to the schedule shown in Fig. 1a. At 10 days after laminectomy, collaterals were rarely observed in the sham mice (Fig. 1b); however, at 10 days after SCI, the number of collaterals sprouting from dorsal CST into the cervical gray matter (C4-C7) was significantly increased (Fig. 1c, e). At 28 days after SCI, the number of collaterals was significantly decreased compared to that at 10 days after SCI, but remained significantly higher than that in the sham mice (Fig. 1d, e). These results suggested that the pruning of collaterals occurred from days 10 to 28 after SCI, consistent with previous observations^8^.

### Nrp1 is upregulated in the motor cortex in the pruning phase

**Fig. 2:**
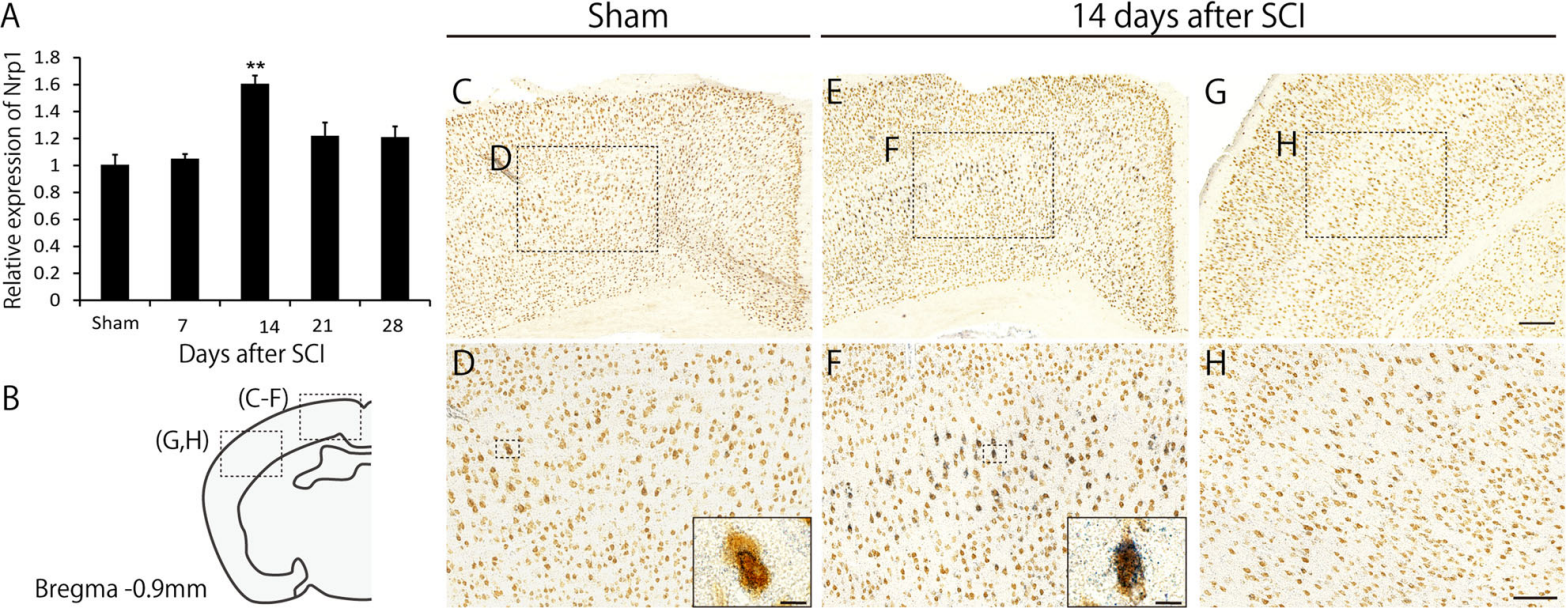
Neuropilin-1 (Nrp1) is upregulated in the motor cortex in the pruning phase. **a** RNA in the motor cortex was extracted at 1, 7, 14, 21, and 28 days after spinal cord injury (SCI) or sham treatment and was subjected to real-time PCR. Nrp1 expression at 14 days after SCI was significantly upregulated compared to that in sham mice. Data are presented as mean ± SEM. *n* = 3, **p < 0.01, one way ANOVA followed by Tukey-Kramer test. **b** Schematic diagrams of brain sections representing the approximate rostrocaudal level relative to bregma, −0.90 mm. **c**, **e** Representative images showing *Nrp1* mRNA signals (blue) counterstained with NeuN (brown) in the motor cortex of sham (**c**) and injured (**e**) mice. In injured mice, more *Nrp1* signals were observed. **d**, **f** Higher magnification images of the boxed regions in (**c**) and (**e**). The inset images in (**d**) and (**f**) are close-up view of the *Nrp1* mRNA positive neurons marked in the boxed area. Scale bar: 10 μm. **g**, **h** In situ hybridization analysis for *Nrp1* mRNA in the somatosensory cortex (**g**) and higher magnification of the boxed region in (**g**) in an injured mouse. Lower intensity of *Nrp1* signals was observed in the somatosensory cortex compared to the motor cortex. Scale bar: 200 μm (**g**), 100 μm (**h**)

We next elucidated the molecular mechanisms underlying the pruning of collaterals after SCI. Studies have shown that semaphorin-plexin signaling is involved in pruning in the developing nervous system^14,15^. We thus focused on this signaling system as a candidate mediator of collateral pruning. By examining the temporal expression of Nrp1, co-receptor of PlexinA^16^, we found that Nrp1 was transiently upregulated in the motor cortex at 14 days after SCI (Fig. 2a). We then carried out in situ hybridization to characterize the localized expression of *Nrp1* mRNA in the brain before and 14 days after SCI. More *Nrp1* signals were observed in layer V pyramidal neurons in the motor cortex compared to control, but not in the somatosensory cortex, 14 days after SCI (Fig. 2b–h). These results suggested that corticospinal neurons started to express Nrp1 when pruning occurs.

### Nrp1 is required for the pruning of collaterals

**Fig. 3:**
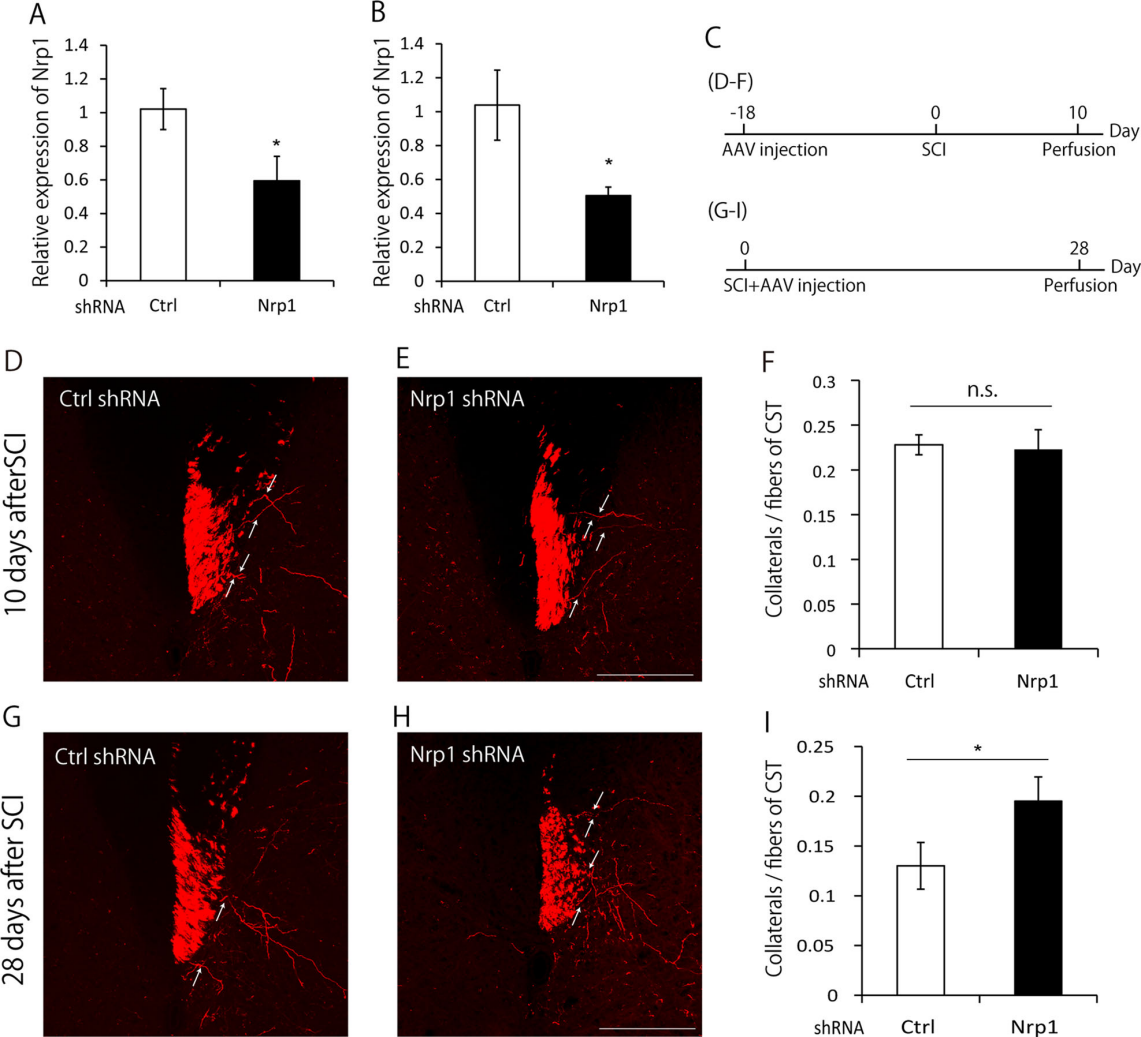
Neuropilin-1 (Nrp1) knockdown suppresses the pruning of collaterals. **a** Quantification of *Nrp1* mRNA by real-time PCR analysis in DIV4 cultured E16 mouse cortical neurons transfected with control shRNA or Nrp1 shRNA. The expression of *Nrp1* was significantly suppressed by Nrp1 shRNA. **b** Quantification of *Nrp1* mRNA by real-time PCR analysis in the motor cortex dissected by laser microdissection at 28 days after the AAV injection. The expression of *Nrp1* was significantly suppressed by AAV expressing Nrp1 shRNA compared to that expressing control shRNA. **c** Experimental time course. To visualize the collaterals at 10 days after spinal cord injury (SCI), AAV was injected into the hindlimb motor area at 18 days before SCI. Then, 10 days after SCI, mice were perfused. To visualize the collaterals at 28 days after SCI, AAV was injected immediately following SCI, and mice were perfused at 28 days after SCI. **d**, **e** Transverse cervical sections showing turbo red fluorescent protein (tRFP)-labeled corticospinal tract (CST) axons (red) in mice infected with AAV expressing control shRNA (**d**) or Nrp1 shRNA (**e**) at 10 days after SCI. White arrows indicate collaterals sprouted from CST. Scale bar: 200 μm. **f** Quantification of the number of collaterals at 10 days after SCI. The number of collaterals in Nrp1 knockdown mice and control mice did not show the significant difference. **g**, **h** Transverse cervical sections showing tRFP-labeled CST axons (red) in mice infected with AAV expressing control shRNA (**g**) or Nrp1 shRNA (**h**) at 28 days after SCI. White arrows indicate collaterals sprouted from CST. Scale bar: 200 μm. **i** Quantification of the number of collaterals at 28 days after SCI. The number of collaterals was higher in Nrp1 knockdown mice compared with control mice. Data are presented as mean ± SEM. *n* = 4 (A), 3 (B), 6–8 (F), 6 (I), *p < 0.05; n.s. no statistical significance, Student’s *t*-test

Since a previous study suggested that Nrp1 was involved in axonal pruning in the developing hippocampus^14^, we assessed whether Nrp1 was also involved in the pruning of collaterals after SCI. We first constructed Nrp1 shRNA vector and AAV1-H1-Nrp1shRNA-CMV-tRFP (turbo red fluorescent protein^17^). We confirmed its knockdown efficiency both in vitro and in vivo by transfection and infection of Nrp1 shRNA, respectively (Fig. 3a, b). We then investigated whether the suppression of Nrp1 expression impaired pruning of collaterals. Since the number of collaterals reached the peak at 10 days after SCI and then decreased by 28 days after SCI (Fig. 1e), we evaluated the number of collaterals at these two time points. To visualize sprouted collaterals from CST fibers at 10 days after SCI, AAV was injected into the hindlimb motor area at 18 days before SCI. Mice were perfused 10 days after SCI (Fig. 3c). We did not observe any significant difference in the number of tRFP-labeled collaterals in mice infected with AAV1-H1-Nrp1shRNA-CMV-tRFP or AAV1-H1-control shRNA-CMV-tRFP (Fig. 3d–f). This suggested that Nrp1 did not affect the sprouting of collaterals after SCI. Next, to visualize the collaterals at 28 days after SCI, AAV was injected immediately following SCI. Mice were perfused 28 days after SCI (Fig. 3c). Suppression of Nrp1 by shRNA led to an increased number of collaterals compared to that of control mice at 28 days after SCI (Fig. 3g–i).

**Fig. 4:**
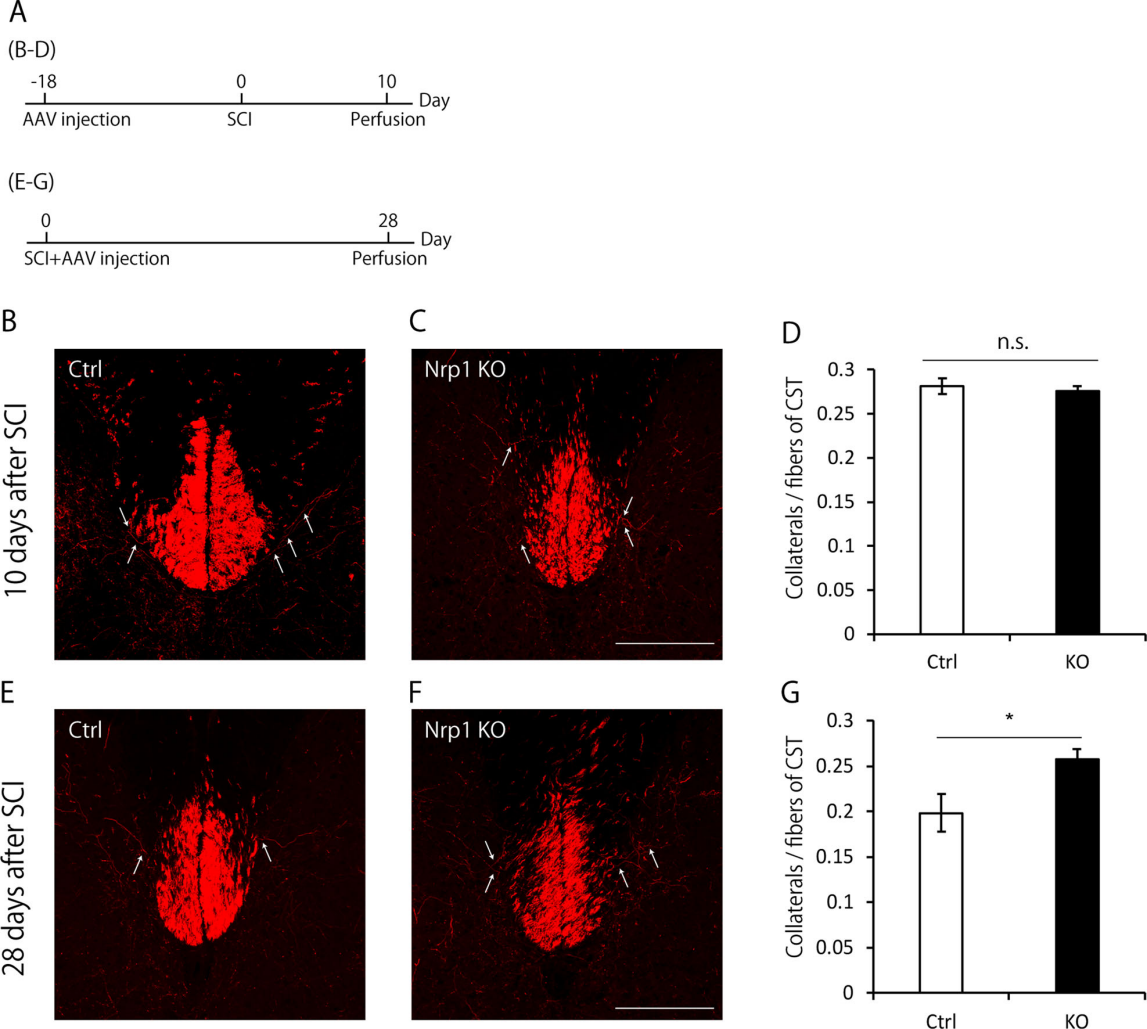
Neuropilin-1 (Nrp1) is required for the pruning of collaterals. **a** Experimental time course. To visualize the collaterals at 10 days after spinal cord injury (SCI), AAV was injected into the hindlimb motor area at 18 days before SCI. Then, 10 days after SCI, mice were perfused. To visualize the collaterals at 28 days after SCI, AAV was injected immediately following SCI, and mice were perfused at 28 days after SCI. **b**, **c** Transverse cervical sections showing tdTomato-labeled corticospinal tract (CST) axons (red) in Ai14 mice (**c**) or Nrp1 floxed mice crossed with Ai14 mice (**d**) infected with AAV expressing Cre recombinase at 10 days after SCI. White arrows indicate collaterals sprouted from CST. Scale bar: 200 μm. **d** Quantification of the number of collaterals at 10 days after SCI. The number of collaterals in Nrp1 knockout mice and control mice did not show the significant difference. **e**, **f** Transverse cervical sections showing tdTomato-labeled corticospinal tract (CST) axons (red) in Ai14 mice (**f**) or Nrp1 floxed mice crossed with Ai14 mice (**g**) infected with AAV expressing Cre recombinase at 28 days after SCI. White arrows indicate collaterals sprouted from CST. Scale bar: 200 μm. **g** Quantification of the number of collaterals at 28 days after SCI. The number of collaterals was higher in Nrp1 knockout mice compared with control mice. Data are presented as mean ± SEM. *n* = 3–4 (**d**), 3 (**g**), *p < 0.05; n.s. no statistical significance, Student’s *t*-test

To further investigate the role of Nrp1 in pruning of collaterals, we injected AAV9-CAMKII-Cre into both sides of the hindlimb motor cortex in Nrp1 floxed mice^18^ crossed with Ai14 (a tdTomato reporter line), in which Cre-mediated recombination induced site-specific and neuron-specific knockout of Nrp1^19^, and the expression of tdTomato. Then, we evaluated the number of tdTomato-labeled collaterals at 10 days and 28 days after SCI according to the same time course with knockdown experiments (Fig. 4a). We found that Nrp1 knockout specifically suppressed the pruning of collaterals as in the shRNA model (Fig. 4b–g). These results suggested that Nrp1 was required for the pruning of collaterals.

### Sema3A expression decreases specifically in LPSNs 14 days after SCI

**Fig. 5:**
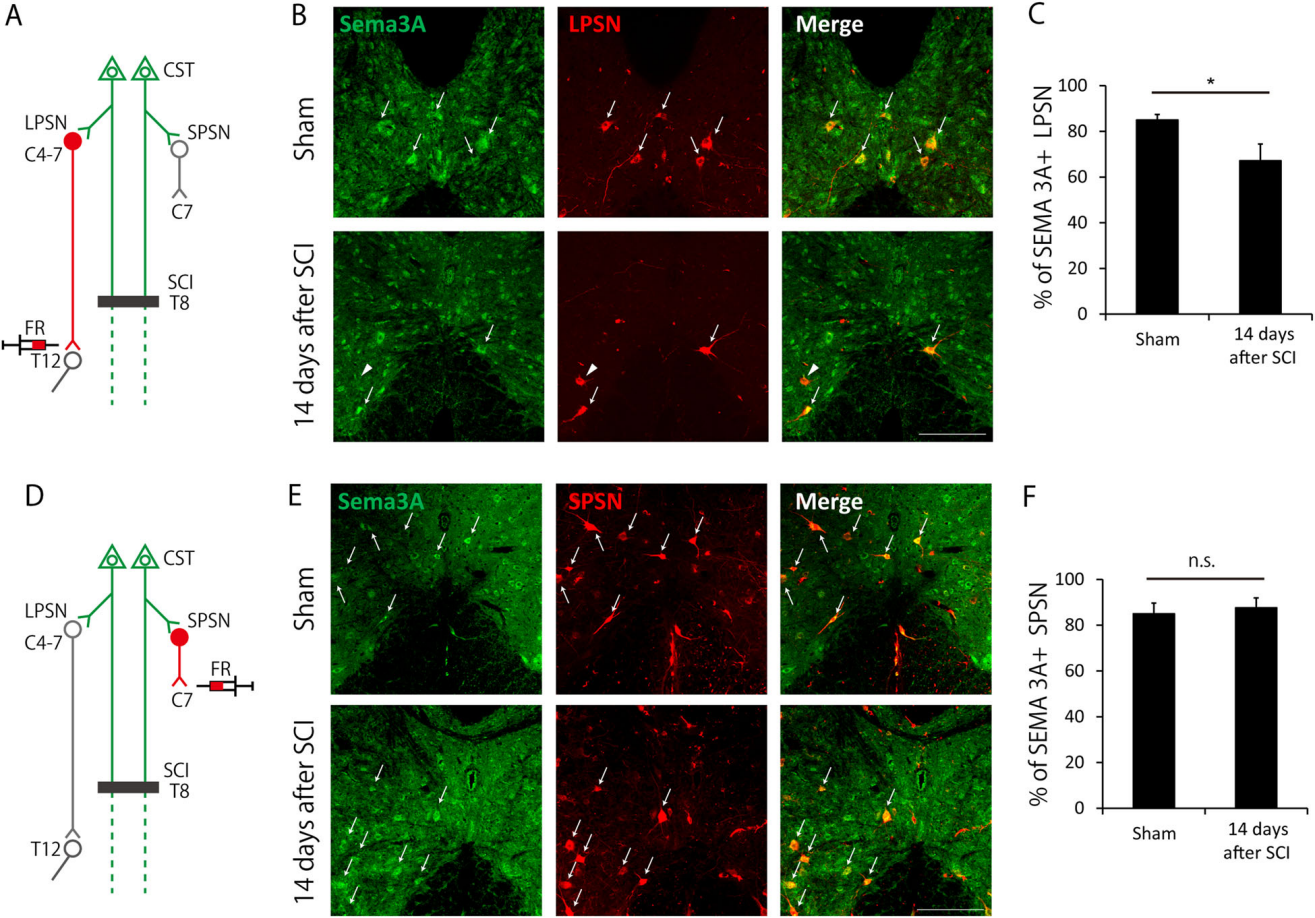
Semaphorin 3A (Sema3A) expression is decreased in long propriospinal neurons (LPSNs) 14 days after spinal cord injury (SCI). **a** Scheme of the experiment in (**b**) and (**c**). To label LPSNs, the retrograde tracer, Fluoro-Ruby (FR), was injected into T12. Immunohistochemistry for Sema3A was then performed. **b** Transverse cervical sections (C4-C7) showing Sema3A and FR-labeled LPSNs in sham (upper) and injured (lower) mice. White arrows indicate LPSNs expressing Sema3A. White arrowheads indicate LPSNs not expressing Sema3A. Scale bar: 200 μm. **c** Quantification of the ratio of LPSNs expressing Sema3A in cervical enlargement. The ratio was decreased after SCI. **d** Scheme of the experiment in (**e**) and (**f**). To label short propriospinal neurons (SPSNs), FR was injected into C7 level spinal cord. **e** Transverse cervical sections (C4-C7) showing Sema3A and FR-labeled SPSNs in sham (upper) and injured (lower) mice. Scale bar: 200 μm. **f** Quantification of the ratio of SPSN expressing Sema3A in cervical enlargement. Data are presented as mean ± SEM. *n* = 7–8 (**c**), 6–7 (**f**), *p < 0.05; n.s. no statistical significance, Welch’s*t*-test (**c**), Student’s *t*-test (**f**)

Previous work has shown that Sema3A binds to Nrp1/plexinA complex and induces growth cone collapse^16^. We, therefore, examined whether Sema3A was expressed in propriospinal neurons in the cervical cord, which is considered a target of collaterals from CST after SCI^6,8^. Propriospinal neurons can be classified into two types, LPSNs and SPSNs, based on the length of their axons^20^. To selectively visualize LPSNs and SPSNs, we injected a fluorescent retrograde tracer, Fluoro-Ruby, into both sides of T12 and C7, respectively, in sham and injured mice (Fig. 5a, d). We then conducted immunohistochemistry for Sema3A on cervical cords of mice 14 days after Fluoro-Ruby injection (Fig. 5b, e). The results revealed that the ratio of LPSN expressing Sema3A in cervical enlargement decreased 14 days after SCI (Fig. 5c), whereas that of SPSN expressing Sema3A did not change (Fig. 5f). These results suggested that the collaterals expressing Nrp1 maintained connections with a subpopulation of Sema3A-negative LPSNs, whereas other collaterals were pruned through Sema3A-Nrp1 interactions.

### Nrp1 is required for motor recovery after SCI

**Fig. 6:**
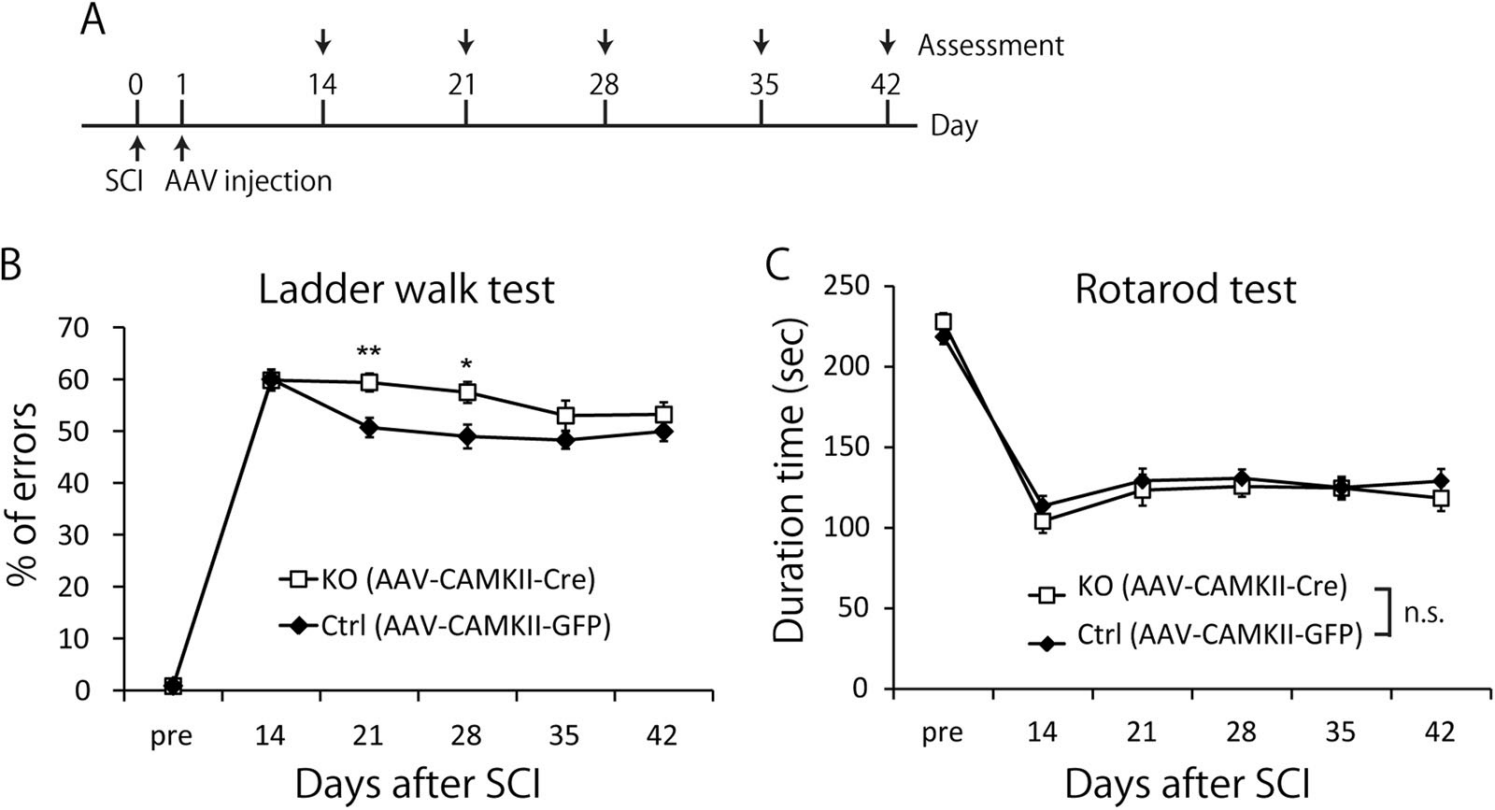
Neuropilin-1 (Nrp1) is required for motor recovery after spinal cord injury (SCI). **a** Experimental time course. At 1 day after SCI, mice were injected with AAV9-CAMKII-Cre or AAV9-CAMKII-EGFP into the hind limb motor area. Motor function was evaluated once a week from 14 to 42 days after SCI. **b** Nrp1 floxed mice injected with AAV9-CAMKII-Cre showed worse motor performance than those injected with AAV9-CAMKII-EGFP at 21 and 28 days after SCI in the ladder walk test. **c** Nrp1 floxed mice injected with AAV9-CAMKII-Cre or AAV9-CAMKII-EGFP did not show significant differences in the rotarod test. Data are presented as mean ± SEM. *n* = 16–17, **p < 0.01, *p < 0.05, two-way repeated ANOVA followed by Bonferroni

Previous studies have shown that pruning of collaterals occurs after SCI^6,8^, but the causal relationship between pruning and motor recovery remains unclear. To determine whether the pruning of collaterals contributes to spontaneous recovery after SCI, two motor tasks, the ladder walk test and the rotarod test, were conducted on Nrp1 floxed mice injected with AAV9-CAMKII-Cre or AAV9-CAMKII-EGFP into the hindlimb motor area. At 21 and 28 days after SCI, the motor performance of Nrp1 floxed mice injected with AAV9-CAMKII-Cre was impaired in the ladder walk test but not in the rotarod test (Fig. 6b, c). Notably, motor impairment was attenuated at 35 and 42 days after SCI. These results demonstrated that Nrp1-mediated pruning of collaterals was necessary for motor recovery.

## Discussion

In the present study, we identified the molecular mechanisms underlying axonal pruning, which is the process of refinement following axonal sprouting and synaptic formation after incomplete SCI. We also showed that the molecule involved in axonal pruning was necessary for motor recovery in the subacute phase of SCI.

Pruning is a regressive process that was originally observed in the developing brain. Pruning is an essential phase which leads to the elimination of unnecessary neural circuits and strengthening of necessary connections to elaborate neural circuits in an activity-dependent manner^10,11^. Recent studies showed that pruning also occurred on collaterals in reestablished neural circuits once these circuits were formed after SCI^6,8^. Nevertheless, the molecular mechanisms and causal relationship between pruning and motor recovery after SCI have hitherto remained unexplored.

Here, we identified Nrp1 as a molecule responsible for the pruning of collaterals. Our study found that Nrp1 was transiently upregulated specifically in the motor cortex (Fig. 2a), whereas, the expression of plexinA family (A1, A2, A3, and A4), which forms a complex with Nrp1 and is involved in signal transduction^21^, was not changed in the motor cortex after SCI (data not shown). Nrp1 is a member of the semaphorin-plexin family that regulates neural circuit formation^21^. Nrp1 is suggested to be associated with axonal pruning in the developing hippocampus^14^. These findings prompted us to investigate the role of Nrp1 in the pruning of collaterals. As a result, we found that suppression and depletion of Nrp1 expression in the motor cortex suppressed the pruning of collaterals (Figs. 3i and 4g). These results suggest that the molecular mechanisms underlying pruning after SCI may be common to those in the developing brain, in line with the concept described previously^22^.

With respect to the ligand of Nrp1, we confirmed Sema3A expression in propriospinal neurons in cervical cords, which formed synapses with collaterals after SCI^6^. We demonstrated that Sema3A expression in LPSNs was decreased after SCI, whereas its expression in SPSNs was maintained (Fig. 5c, f). This result may explain the molecular basis for selective pruning after SCI as described in a previous study, suggesting that the collaterals connected with SPSNs were eliminated dominantly because the axons of SPSNs did not bridge the lesion^6^. Our results suggest a model whereby Sema3A is downregulated only in LPSNs which are integrated into functional compensatory neural circuits to avoid pruning of collaterals through Sema3A-Nrp1 interaction. Indeed, previous studies showed that Sema3A was downregulated by electrical activity^23,24^. Therefore, we consider that neural activity in LPSNs caused by the inputs from the collaterals may downregulate the expression of Sema3A after SCI. A recent study showed that both excitatory and inhibitory LPSNs exist in the cervical cord, and excitatory LPSNs could be further classified into several subtypes in accordance with their progenitor domain origins^25^. This result raises the possibility that pruning may selectively occur based on the criteria mentioned above.

Previous studies demonstrated that microglia are involved in the pruning of axons and synapses through the classical complement cascade^26^. In our study, we found that pruning of collaterals was suppressed with neuron-specific Nrp1 knockout by injecting AAV-CAMKII-Cre into Nrp1 floxed mice (Fig. 4g). This result suggests that Nrp1-mediated pruning of collaterals can occur independent of microglia.

Our results highlighted the functional significance of pruning post-SCI. Virus-mediated genetic deletion of Nrp1 specifically in the motor cortex after SCI impaired functional recovery in the ladder walk test but not in the rotarod test (Fig. 6b, c), suggesting that Nrp1-mediated pruning was required for motor recovery after SCI. The task-dependent difference in recovery may reflect the extent of involvement of CST in the motor performance required for each test. Specifically, CST-mediated precise hindlimb movement is required in the ladder walk test^27^. It should be noted that the impairment of motor recovery by Nrp1 knockout disappeared at 35 and 42 days after SCI (Fig. 6b). This could have been due to at least two possibilities. One is that other molecules may be involved in the pruning of collaterals at timepoints after 35 days post-SCI. The other is that compensatory mechanisms may be enacted when Nrp1-mediated pruning is insufficient. Regardless, our findings may partly explain why enhanced sprouting by the manipulation of the relevant molecules did not necessarily contribute to motor recovery after SCI in a previous study^28^. Our results suggest that reconstruction of appropriate neural circuits is also critical for motor recovery.

These findings may lead to potential novel treatments for SCI. Specifically, they suggest that promoting the pruning of collaterals from CST fibers can facilitate motor recovery by the refinement of compensatory neural circuits. Moreover, as the pruning of collaterals also occurs after brain injury^29^, this strategy has the potential to be applied to the treatment of brain injury. Further research is needed to reveal the precise mechanisms underlying selective pruning and effective ways to promote the elaboration of neural circuits by pruning. These efforts will contribute to the development of new therapeutic strategies for motor recovery after neurotrauma.

## Materials and methods

### Animals

Adult C57BL/6J female mice (age: 7–8 weeks, Japan SLC, Inc.), Nrp1 floxed female mice (age: 7–9 weeks, B6.129(SJL)-Nrp1^tm2Ddg^/J from the Jackson Laboratory), in which exon 2 of the *Nrp1* allele was flanked by loxP sequences bilaterally^18^, and Ai14 female mice (age: 7–9 weeks, B6.Cg-Gt(ROSA)26Sor^tm14(CAG-tdTomato)Hze^/J from the Jackson Laboratory) were used in this study. All experimental procedures were approved by the Institutional Ethics Committee of Osaka University and complied with the Osaka University Medical School Guidelines for the Care and Use of Laboratory Animals.

### Spinal cord injury

The surgical procedures were slightly modified from a previous study^12^. Briefly, mice were anesthetized with a mixture of 0.5 mg/mL Vetorphale (Meiji Seika Pharma), 0.4 mg/mL Dormicum (Roche), and 0.03 mg/mL Domitor (Orion Pharma). Thoracic level 8 (T8) laminectomy was performed, and a dorsal hemisection of the spinal cord at a depth of 1.0 mm was conducted with a surgical microknife.

### Behavioral tests

#### Rotarod test

The rotarod was used to assess motor recovery in rodents after SCI^30,31^. For the rotarod test, animals were placed on a rotating rod (diameter 30 mm) that accelerated from 0 to 50 r.p.m. within 5 min. Mice were trained three times a day for 3 days before SCI. The total time was recorded until the mouse fell off the rod or gripped and spun around two times as described previously^32^. The baseline value was scored as the mean of three trials 1 day before SCI (third training session).

#### Ladder walk test

The ladder walk test was used to assess skilled walking, precise hindlimb placing, and interlimb coordination^27^. The animals were placed on a 1-m runway comprising randomly spaced metal rungs. Foot faults for each hindlimb were counted during continuous steps. The setup of rungs was changed every week to rule out learning effects. The baseline value was assessed 1 day before SCI.

### Construction of plasmids and adeno-associated virus purification

The procedures were slightly modified from a previous study^33^. Briefly, Nrp1 and control shRNAs, tRFP, Cre recombinase, and EGFP cDNAs were cloned into plasmid pAAV-MCS (Stratagene). AAV 293cells were cultured in sixty 10-cm diameter dishes in Dulbecco’s modified Eagle medium (DMEM, Thermo Fisher Scientific) with 10% fetal bovine serum (FBS, Thermo Fisher Scientific). After cells were 70–80% confluent, 300 µg of Rep/Cap plasmid, 600 µg of Adenovirus-helper plasmid, and 300 µg of pAAV-MCS were transfected into cells. Five days after transfection, cells were washed in phosphate-buffered saline (PBS) and the cell suspensions were collected. After five freeze/thaw cycles of the cell suspensions, Benzonase Nuclease (Sigma-Aldrich) was added into the suspensions and centrifuged at 8000 rpm, 4 °C for 40 min. Ammonium sulfate was then added into the supernatants, which were centrifuged at 8000 rpm, 4 °C for 40 min, and filtered with 0.45 µm filter (Merck) to collect the fraction containing AAV. After establishing a gradient of AAV solution by OptiPrep (Axis-Shield), the gradient was ultracentrifuged at 36,000 rpm, 16 °C for 16 h. AAV solution was then concentrated with PBS by Amicon ultra-15 (Merck) until the volume was ~250 μL.

### Anterograde tracing of hindlimb corticospinal tract

The descending CST fibers were labeled with the anterograde tracer, biotinylated dextran amine (BDA; MW, 10,000; 10% BDA in PBS; Invitrogen), AAV1-H1-shRNA-CMV-tRFP, or AAV9-CAMKII-Cre. Tracer injection procedures were performed as described previously^12^. Briefly, the animals were anesthetized and placed in a stereotaxic frame. The corresponding skull area was exposed. BDA was injected at 10 days before the animals were perfused. 0.8 µL/site BDA was bilaterally infused into the hindlimb motor area of wild-type mice (coordinates from bregma: 1.0 mm posterior/0.8 mm lateral, 1.0 mm posterior/1.3 mm lateral, all at a depth of 0.6 mm). AAV1-H1-shRNA-CMV-tRFP or AAV9-CAMKII-Cre was injected at 28 days before the animals were perfused. 0.4 µL/site AAV1-H1-shRNA-CMV-tRFP was infused into the right hindlimb motor area of wild-type mice (coordinates from bregma: 0.8 mm posterior/0.8 mm lateral, 0.8 mm posterior/1.3 mm lateral, 1.3 mm posterior/0.8 mm lateral, and 1.3 mm posterior/1.3 mm lateral, all at a depth of 0.6 mm). 0.4 µL/site AAV9-CAMKII-Cre was bilaterally infused into the hindlimb motor area of Ai14 mice or Nrp1 floxed mice crossed with Ai14 mice (coordinates from bregma: 1.0 mm posterior/1.0 mm lateral at a depth of 0.6 mm). These tracers were infused using a glass capillary attached to a microsyringe (Ito). After injection, the pipette tip was left at the injection site for 2 min before withdrawal to prevent tracer backflow.

### Hindlimb motor area specific knockout of Nrp1

To genetically deplete Nrp1 expression in neurons, 0.4 µL/site AAV9-CAMKII-Cre or AAV9-CAMKII-EGFP (cotrol) was bilaterally infused into the hindlimb motor area of Nrp1 floxed mice (coordinates from bregma: 0.8 mm posterior/0.8 mm lateral, 0.8 mm posterior/1.3 mm lateral, 1.3 mm posterior/0.8 mm lateral, and 1.3 mm posterior/1.3 mm lateral, all at a depth of 0.6 mm) at 1 day after SCI.

### Retrograde tracing of propriospinal neurons

The long propriospinal neurons (LPSNs) and short propriospinal neurons (SPSNs) were labeled with the retrograde tracer Fluoro-Ruby (MW, 10,000; 10% Fluoro-Ruby in PBS; Invitrogen) 2 weeks before the animals were perfused. Tracer injection procedures were modified slightly from a previous study^34^. Briefly, the animals were anesthetized and placed in a stereotaxic frame. To label the LPSNs and SPSNs, 0.6 µL/site or 0.4 µL/site Fluoro-Ruby was bilaterally infused into the spinal cords at vertebral level T12 or C7 (lateral, ±0.8 mm, depth of 0.9 mm), respectively.

### Immunohistochemistry

The animals were deeply anesthetized and transcardially perfused with 4% paraformaldehyde (PFA) in 0.1 M phosphate buffer. Following perfusion, the brain and spinal cord were postfixed in 4% PFA at 4 °C overnight and subsequently transferred to 30% sucrose in PBS. The tissues were embedded in Tissue-Tek O.C.T. Compound (Sakura Finetek). The spinal cords were cut into 30-µm-thick coronal sections using a cryostat and placed on MAS-coated glass slides (Matsunami). For immunohistochemistry, the sections were immersed three times in PBS and blocked with 5% bovine serum albumin/0.1% Triton X-100/10% normal donkey serum/PBS for 1 h. Subsequently, the sections were incubated with primary antibody overnight at 4 °C. Goat anti-semaphorin-3A (SEMA3A) (1:100; Santa Cruz Biotechnology)^35^ was used as the primary antibody. The sections were subsequently washed three times with PBS, followed by incubation with a secondary antibody for 1 h at room temperature in the dark. Donkey-anti-goat IgG Alexa 488 (1:400; Invitrogen) was used as the secondary antibody. For BDA labeling, the sections were immersed three times in PBS and incubated in 0.3% Triton X-100/PBS for 2 h, followed by incubation with Alexa Fluor 488-conjugated streptavidin (1:400; Invitrogen) for 1 h at room temperature. All images were acquired with a confocal laser-scanning microscope (Olympus FV1200).

### In situ hybridization

In situ hybridization and histology were performed as previously described^36^. The primer sequences for *Nrp1* probe were as follows: forward, 5′-AATGAATGGCTCCAAGTG GAC-3′; reverse, 5′-GATCCAGGGTCTTAAGCACAT-3′. All images were acquired with a fluorescence microscope (Olympus IX83).

### Cortical neuron culture and nucleofection

E16 mouse cortices were digested in 0.25% trypsin (Thermo Fisher Scientific) supplemented with DNase (1:1000, Sigma) for 15 min at 37 °C. Cells were dissociated in DMEM/F12 (Thermo Fisher Scientific) containing 10% FBS (Thermo Fisher Scientific). Cells were then washed and resuspended in 100 μL of Mouse Neuron Nucleofector Solution (Lonza) containing 3 μg of Nrp1 shRNA or control shRNA (points-mutated Nrp1 shRNA) vector. The shRNA sequences were as follows: Nrp1, 5′-AGAGAAGCCAACCATTATA-3′; control, 5′-AGAGAAGACAGCCCTTATA-3′^37^. After electroporation using a Nucleofector kit (Lonza), the cells were immediately mixed with 500 μL of DMEM/F-12 containing 10% FBS, and the cells were plated onto poly-l-lysine-coated dishes and cultured at 37 °C in the presence of 5% CO_2_. The medium was exchanged with DMEM/F-12 (Thermo Fisher Scientific) containing 2% B27 supplement (Thermo Fisher Scientific) 12 h later.

### Laser microdissection

Fresh brains were immediately frozen on dry ice 28 days after bilateral injections of AAV vector. The tissues were then cut into 10 μm-thick sections and mounted on glass slides with Poly(p-phenylene sulfide) (PPS) foil (Leica Microsystems). After toluidine blue staining, the motor area was dissected bilaterally with an LMD 7000 (Leica Microsystems) and transferred into microcentrifuge tube caps filled with 50 μL of TRIzol reagent (Thermo Fisher Scientific).

### Quantitative polymerase chain reaction

Cells and the motor cortex of mice were dissected and homogenized in TRIzol reagent (Thermo Fisher Scientific). Isolated total RNA was purified with an RNeasy Micro kit (QIAGEN). The complementary DNA (cDNA) was synthesized with a High Capacity cDNA Reverse Transcription kit (Applied Biosystems). Primer sequences for *Nrp1* were as described previously^38^. Real-time polymerase chain reaction (PCR) analysis was conducted using SYBR Green Master Mix (Thermo Fisher Scientific). The expression of *Nrp1* was normalized to *Mrpl32*^38^. The reaction and subsequent quantification were conducted using QuantStudio 7 Flex Real-Time PCR System (Applied Biosystems).

### Quantification of collaterals and CST fibers

The quantification method was slightly modified from a previous study^6^. Briefly, to assess the number of sprouted collaterals from hindlimb CST, collaterals entering the gray matter from the dorsal funiculus in the cervical cord (segments C4 to C7) were counted in 30 transverse sections. We then divided the total number of collaterals by the number of fibers in dorsal CST at C3 level to normalize tracing differences among individual animals. The number of fibers was determined with ImageJ software (US NIH).

### Quantification of Sema3A-positive propriospinal neurons

The quantification method was slightly modified from a previous study^34^. Briefly, to calculate the ratio of long and short propriospinal neurons expressing Sema3A, retrogradely-labeled neurons located in C4 to C7 were counted until the number of cells reached 30 per mouse. Results were expressed as a ratio of the number of double-labeled neurons in 30 assessed neurons.

### Statistical analysis

All data are presented as mean ± SEM. Statistical analyses were performed using GraphPad Prism 7 (GraphPad Software). Results were analyzed using Student’s *t*-tests, Welch’s *t*-test, one-way ANOVA followed by Tukey-Kramer tests, and two-way ANOVA followed by Bonferroni tests. *p* values of <0.05 were considered statistically significant.
